# Rapid Resolution of Pulmonary Embolism and Pacemaker Lead Thrombus With Low-Dose Systemic Thrombolytic Infusion

**DOI:** 10.7759/cureus.103295

**Published:** 2026-02-09

**Authors:** Rebecca A Pilkington, Jared W Berger, Shilpa Jasti, Stephen Miller, Ambika Anand

**Affiliations:** 1 Department of Emergency Medicine, Virginia Commonwealth University Medical Center, Richmond, USA; 2 Department of Emergency Medicine, Virginia Commonwealth University School of Medicine, Richmond, USA

**Keywords:** acute pulmonary embolism, deep vein thrombosis (dvt), intravenous thrombolytic therapy, pacemaker lead thrombus, ultrasound (u/s)

## Abstract

Pacemaker lead-associated thrombosis is a rare but clinically significant condition that can lead to embolic complications and hemodynamic instability. Management of pacemaker lead-associated thrombi can range from anticoagulation or systemic thrombolysis to catheter-based extraction and surgical removal. In this case, an 80-year-old male patient presented to the Emergency Department (ED) with acute exertional dyspnea and was found to have extensive left lower extremity deep vein thrombosis (DVT), bilateral segmental pulmonary embolism (PE), and a thrombus adhered to the right atrial pacemaker lead. He was treated with low-dose intravenous alteplase infusion as well as heparin in the intensive care unit, leading to rapid thrombus resolution. He was subsequently transitioned to a direct-acting oral anticoagulant (DOAC) and discharged in stable condition. Treatment for pacemaker lead thrombi is not protocolized, given the dependence on various patient-related and situational factors. This case highlights the importance of early recognition and management of pacemaker lead-associated thrombosis and demonstrates a novel treatment approach in the form of low-dose alteplase infusion. Low-dose thrombolytics may be a safer and less invasive treatment modality in hemodynamically stable patients with a high clot burden.

## Introduction

Pacemaker lead-associated thrombi can result in embolic complications, pacemaker dysfunction, and hemodynamic instability. While permanent pacemakers are widely utilized for the management of cardiac conduction disorders, their intracardiac leads can serve as a nidus for thrombus formation due to foreign material exposure, endothelial disruption, and patient-specific prothrombotic factors. The incidence of pacemaker lead thrombi is not well established, with an incidence of 1.4% in a large study that reviewed over 70,000 transthoracic echocardiograms and a smaller study of patients undergoing ablation [1]. Moreover, some cases remain asymptomatic and are detected incidentally during work-up of various differential diagnoses or post-mortem, upwards of 30-40% of patients with pacemaker/Implantable Cardioverter-Defibrillator (ICD) in a European autopsy study [2]. These thrombi, depending on their location within the heart, can lead to arterial emboli, pulmonary emboli (PE), and right heart failure or strain, requiring timely diagnosis and intervention. 

The management of pacemaker lead-associated thrombi is highly debated, with treatment strategies ranging from anticoagulation and systemic thrombolysis to catheter-based extraction and surgical removal. Systemic thrombolysis has been successfully utilized in patients, particularly those in whom surgical intervention poses a high risk [3]. Additionally, a multimodal approach incorporating surgical, catheter-based, and pharmacologic therapies has been utilized in complex cases of thromboembolism-in-transit with PE [4]. The therapy is often guided by thrombus size, mobility, embolic potential, and patient-specific risk factors. However, there are few high-impact or large population studies completed to date to establish standardized guidelines for optimal management in high-risk or atypical cases. 

In this report, we present the case of an 80-year-old male patient with extensive thrombotic burden, including PE, deep vein thrombosis (DVT), and a thrombus adhered to his right atrial pacemaker lead. This case highlights the importance of early recognition of these various clots and explores systemic lytic infusion as a non-invasive treatment option for PE in patients with significant clot burden including those with pacemaker lead-associated thrombi. 

## Case presentation

An 80-year-old male patient presented to the emergency department (ED) for a sudden onset, worsening exertional dyspnea for two days. His medical history included atrial fibrillation for which he had a left atrial appendage occlusion (LAAO) device placed about nine months prior to presentation, and sick-sinus syndrome for which he had a dual-chamber pacemaker placed years earlier. Additional history included monoclonal gammopathy of undetermined significance and prostate cancer, for which he had undergone prostatectomy and radiation treatment. At baseline, before the onset of symptoms, he was able to take short walks with a cane and complete daily tasks, though he was now unable to walk to the end of the driveway or stand in the kitchen to cook a meal. He was not on any outpatient anticoagulation despite a CHA_2_DS_2_-VASc or congestive heart failure, hypertension, age ≥75 (doubled), diabetes, stroke (doubled), vascular disease, age 65 to 74 and sex category (female) score of three due to his history of recurrent falls [5]. 

ED vitals at rest demonstrated hemodynamic stability with normal blood pressure, heart rate, respiratory rate, and normal oxygen saturation on room air. The patient’s oxygen saturation during ambulation on home pulse oximetry, however, was reportedly as low as 78%. On exam, the patient was not in acute distress and breathing comfortably but with bilateral lower extremity pitting edema more prominent on the left. 

**Figure 1:**
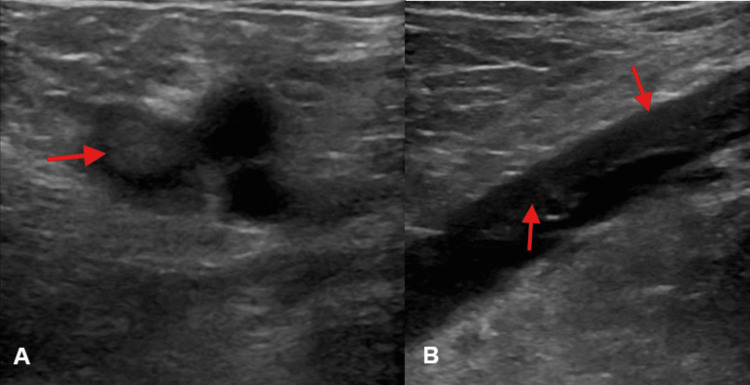
Ultrasound images (A) Deep vein thrombosis (red arrow) on ultrasound of the left common femoral vein at the saphenofemoral junction; (B) Deep vein thrombosis (red arrows) on sagittal view ultrasound of the left popliteal vein.

Laboratory workup showed an undetectable troponin and normal B-type natriuretic peptide. He was found to have extensive acute left lower extremity DVT on ultrasound (Figures 1A, 1B).

**Figure 2:**
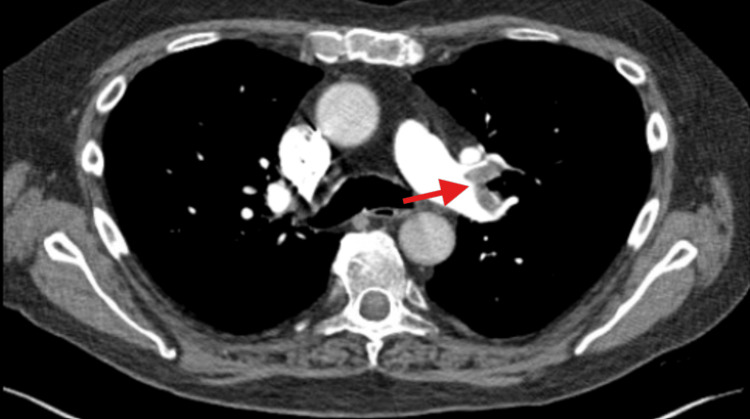
CT angiogram scan of the chest demonstrating a large left main pulmonary artery embolism (red arrow) CT: computed tomography.

Bilateral segmental PE was observed on chest computed tomography (CT) (Figure 2).

**Figure 3:**
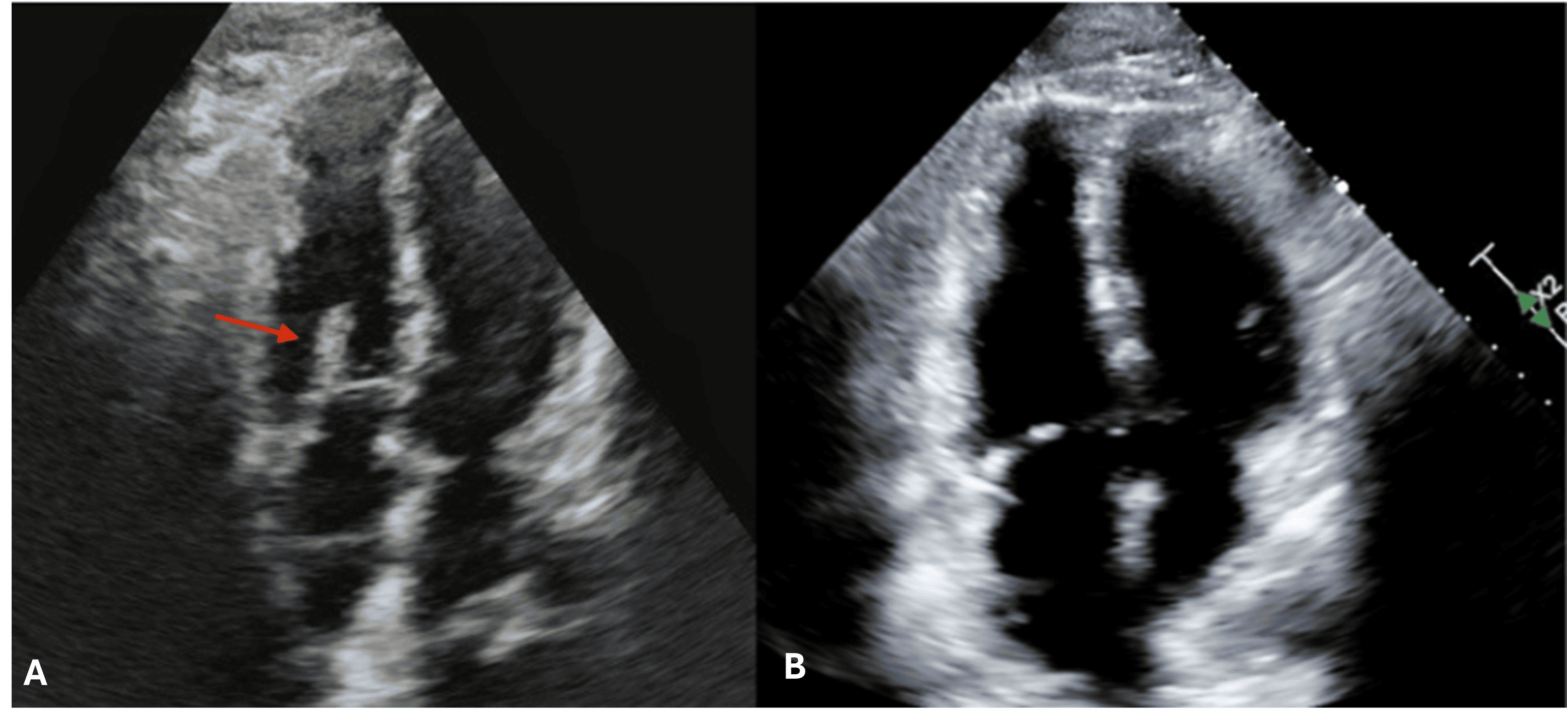
Transthoracic echocardiograms (A) Transthoracic echocardiogram with intracardiac thrombus (red arrow) prior to lytic infusion; (B) Transthoracic echocardiogram with no apparent thrombus after lytic infusion.

The right to left ventricular ratio on chest CT was 1:1 , suggestive of mild right heart strain, prompting activation of our institution’s pulmonary embolism response team (PERT) alert. A bedside echocardiogram was performed, which demonstrated an approximately 5 cm clot-in-transit adhered to the patient’s right atrial pacemaker lead but no evidence of right heart strain, with the right ventricle smaller than left ventricle (Figure 3A). 

His Pulmonary Embolism Severity Index (PESI) score was 120, placing the patient in the high-risk category. But it was worth noting that he scored points only for age, sex, and cancer history [6]. The patient was in the intermediate-low risk category for PE as per the European Society of Cardiology guidelines [7]. 

The patient was started on a heparin infusion and admitted to the intensive care unit (ICU) for monitoring. He was evaluated by an interventional radiologist who felt that his clinical presentation was reassuring and that embolectomy may carry more risk than benefit. The interventional radiologist suggested low-dose alteplase infusion to expedite clot degradation with escalation to mechanical thrombectomy in case the patient developed any signs of hemodynamic or respiratory instability. Overnight, the patient received 1 mg/hr of alteplase for 12 hours total in addition to the heparin infusion. He remained hemodynamically stable throughout the treatment. The ICU ordered hypercoagulation work-up including antithrombin, lupus antibody, protein C/S activity, factor V Leiden, etc. However, the results did not return until the time of his post-discharge follow-up visits. 

On hospital day two, the formal transthoracic echocardiogram demonstrated complete dissolution of his pacemaker lead thrombus and normal right heart systolic function (Figure 3B). The patient developed hemorrhagic bullae on his upper extremity but no other bleeding complications. He was transitioned to a direct-acting oral anticoagulant (DOAC) on hospital day three and discharged home with improvement in his exertional dyspnea, but did not have repeat imaging to re-evaluate pulmonary arterial clot burden. 

The discharging team ordered primary care follow-up within a week of discharge both for general follow-up and for pending anticoagulation work-up. The lupus anticoagulant test returned positive and his primary doctor placed a hematology referral. Hematology informed the patient of possible interference of heparin administration at the time of the initial test with accurate results and repeated this lab three months later, which remained positive. The patient was formally diagnosed with antiphospholipid syndrome and transitioned to warfarin approximately four months after discharge. 

## Discussion

Pacemaker lead-associated thrombi have been well-detailed in various case reports spanning decades, though there is no consensus for a single best treatment approach, with options including thrombolysis, anticoagulation, endovascular retrieval, and surgical extraction. Though pacemaker lead-associated thrombi are seemingly rare, data are variable regarding their incidence, given that a portion are incidentally found and asymptomatic. In a study of 1086 patients with intracardiac devices who had echocardiograms, the incidence was found to be 1.4%, though this ranges from 0.6% to over 3% in other studies [1]. Many are found post-mortem on autopsy in up to 30-40% of patients with pacemaker/ICD [2]. 

Many case reports detail anticoagulation with heparin or warfarin for dissolution of the clot with subsequent continuation of anticoagulants [8,9]. Others demonstrate surgical extraction of leads, thrombus, or both in cases of large thrombi, valve obstruction, pacemaker failure, or shock [10,11]. Few report endovascular thrombectomy which carries a higher mortality [12]. The unique aspect of this case is the extent of clot burden given presence of DVT and bilateral PE in conjunction with pacemaker lead thrombus. This prompted the subsequent use of a low-dose thrombolytic infusion as initial therapy after admission, which is seemingly less well studied in literature and uncommon on review of published case reports. 

Treatment regimen for thrombolytic therapy, including dosage and medication selection, is not well agreed upon, and there is no guideline for the current treatment of pacemaker lead-associated thrombi. The goal of any therapeutic modality is to balance risk and benefit, which ranges widely between studies, many of which focus on PE treatment rather than pacemaker lead thrombi. The initial Pulmonary Embolism International THrOmbolysis (PEITHO) trial focused on thrombolytic therapy for PE, demonstrating a reduction in hemodynamic collapse or decompensation, though the treatment was accompanied by an increased risk of severe hemorrhage [13]. 

Other metanalyses have demonstrated that low-dose thrombolytics, such as alteplase, have similar efficacy to standard dosing or are superior to heparin alone. They also have a dose-dependent relationship with bleeding, making them a potentially safer option for patients at higher risk for hemorrhagic complications [14,15]. However, it is difficult to extrapolate these studies to this case, as the thrombotic burden was higher and it included pacemaker lead thrombus, which is by far less studied on a larger scale. 

The SEATTLE II study, while focusing on PE, did use a similar dosing of tissue plasminogen activator at 1 mg/hr for 12 hours with clinical improvement and low hemorrhagic complications [16]. Though the study focused on treatment for a PE, the regimen was similar to that used for our patient. A future study using this regimen would be more applicable to creating guidelines for treatment of pacemaker lead thrombi, though given the extensive thrombotic burden of our patient, including a PE, there may be some generalizability when using low-dose thrombolytics. 

Most of the literature for pacemaker lead thrombi are case reports. There are several early reports of thrombolytics such as streptokinase and recombinant tissue plasminogen activator (rTPA) use; however, these were used in patients who had failed initial anticoagulant therapy with heparin or warfarin prior to starting thrombolytics [17-19]. The rTPA case reports both discussed using a thrombolytic regimen spanning several days rather than a one-time dose with 40mg/day for five days after an initial lower dose bolus of 10mg [17,19].

In our case, the decision to use thrombolytics was made early as recommended by interventional radiology, shortly after admission, with no significant complications. While the patient developed a hemorrhagic bulla on the left upper extremity, there were no other hemorrhagic complications. Dissolution of the lead thrombus was accomplished using only 1 mg/hr for 12 hours. This raises the question of the best method for initial treatment, choice of medication, when to transition to thrombolytics, and what dose of thrombolytics to use. More importantly, these decisions are seemingly dependent on the patient’s individualized clinical presentation, including risk factors, age, cardiac history, bleeding risk, degree of thrombotic burden, current anticoagulation status, and cardiovascular stability. Our patient had extensive clot burden in the left leg, right atrial lead, and bilateral segmental pulmonary arteries. Despite this clot burden, he remained hemodynamically stable with a functional pacemaker and no outpatient anticoagulation before presentation, making him a reasonable candidate for this therapy. 

As previously detailed, there is no agreed upon evidence-based guideline for pacemaker lead thrombi despite significant literature related to PE, though there may be a place for patients who are otherwise poor candidates for surgical intervention or at high risk for bleeding. In a case report of a 74-year-old patient with brain metastasis within 30 days post-op of craniectomy and right atrial thrombus, low-dose TPA was given with no hemorrhagic complications [20]. The largest study, completed in 1999, included 38 patients with right heart thrombi with associated PE who received thrombolytics, with no statistically significant change in mortality between those receiving thrombolytics vs those not receiving them [21]. There was a difference when patients were treated earlier as decompensation to cardiac arrest worsened outcomes, leading investigators to conclude that thrombolytics may have a role in bridging patients to further intervention by preventing that decompensation [21]. A smaller and more recent study, done in 2019, with five patients, found complete resolution of right heart thrombi following half-dose alteplase with no hemorrhagic complications and clinical improvement [22]. Given the variable and limited data, low-dose thrombolytic therapy is not a currently accepted evidence-based practice for pacemaker lead thrombi, though they may have some efficacy and present with a lower bleeding complication rate in select patients, such as this case. However, patient-dependent factors, as discussed, should be considered extensively when considering this therapy. 

Individual patient factors also raise the question of the utility of prophylactic anticoagulation in patients with pacemakers who have a high risk for thrombotic or embolic events. Our patient was noted to have a history of atrial fibrillation, placing him at a greater risk of embolic events. He was not on anticoagulation therapy prior to presentation, due to the risk of intracranial hemorrhage in the setting of recurrent falls. His cardiologist opted for a LAAO device; however, this device does not prevent lead thrombosis. His risk otherwise for DVT and PE was relatively low, but did having pacemaker leads increase his risk enough to warrant prophylactic anticoagulation? Several cases detail initially silent thromboses, which were identified postmortem after cardiac arrest [23]. Silent thrombi that go undiagnosed are common, and are sometimes incidentally diagnosed. However, they can progress to rare but serious life-threatening complications [24]. In some cases, patients presented with months of nonspecific symptoms that were misconstrued as chronic bronchitis or heart failure with thrombi missed on transthoracic echocardiography only to be found later with transesophageal echocardiography during subsequent admissions [25]. 

While there are no active studies for thrombolytic therapy for pacemaker lead thrombi, there is an active randomized, double blind, multinational trial for dose-reduced thrombolysis for PE (PEITHO-3 trial) that could lay a framework for future management and research for pacemaker lead thrombi [26]. Further clinical research is therefore necessary to better standardize therapeutic guidelines for pacemaker lead thrombi. 

## Conclusions

This independent case spotlights the potential role for prophylactic anticoagulation in prevention of thrombotic events such as PE and pacemaker lead-associated thrombosis in high-risk patients with similarly elevated CHA₂DS₂-VASc scores, and the use of low-dose thrombolytic infusions for clot dissolution. Low-dose thrombolytics could be considered a primary option for treatment in clinically stable patients with low risk for hemorrhagic complications to promote clot dissolution with less invasive techniques. However, the generalizability of this case is limited as it is an isolated case. Further clinical investigation is needed for better evidence-based practices regarding treatment options and the role of prophylaxis, though low-dose thrombolytics may be worth considering in the appropriate patient population. 
